# How does the Internet enhance the subjective well-being of elderly individuals in China?

**DOI:** 10.3389/fpsyg.2022.1036169

**Published:** 2022-10-18

**Authors:** Xuebing Dong, Shunjie Meng, Danbo Chen

**Affiliations:** ^1^China Academy of West Region Development, Zhejiang University, Hangzhou, China; ^2^School of Economics, Zhejiang University, Hangzhou, China; ^3^School of Urban and Regional Science, Shanghai University of Finance and Economics, Shanghai, China

**Keywords:** Internet use, elderly individuals, SWB, physical satisfaction, life satisfaction

## Abstract

Although several studies have explored the relationship between the Internet and elderly individuals, little is known about whether and how the Internet affects elderly individuals’ subjective well-being (SWB) from multiple perspectives. This study examines the effects of the Internet on physical satisfaction and life satisfaction and explores the potential mechanisms by which the Internet produces its effects on elderly individuals. Using nationally representative data from the China General Social Survey (CGSS), this study finds that the Internet has a significant positive impact on physical satisfaction and life satisfaction of the elderly in China. The mechanism analysis shows that the Internet can improve the level of health insurance participation, which we interpret as potential mechanisms through which the Internet positively affects physical satisfaction among elderly individuals. Correspondingly, the Internet affects life satisfaction of elderly individuals by influencing social networks. Further heterogeneity tests find that the effect is stronger for urban areas, male and high human capital samples. This study highlights the important micro effects of the Internet and provides a reference for exploring the mechanism of the Internet affecting SWB.

## Introduction

Population aging in both developed and developing countries has become increasingly urgent and complicated. In particular, China has the most senior citizens worldwide, and its population is also expanding at a quick rate (Tian, 2016). More than 18% of China’s population is over the age of 60, and the elderly are predicted to make up one-third of the country’s population by 2050, according to the findings of China’s seventh national census. However, the World Happiness Report in 2022 shows that China ranks 72nd out of 146 countries and regions in terms of people’s happiness, which means that rapid economic growth has not brought more happiness to the elderly. With the advent of the digital economy, the Internet is changing the traditional patterns of life and social relationships of individuals in unprecedented ways in China (Wong et al., 2014; Schehl et al., 2019), especially after the COVID-19 pandemic. On the one hand, the Internet is rapidly overtaking other forms of communication as the primary medium for individuals. On the other hand, the Internet is a large reservoir of knowledge and resources that may considerably improve and broaden people’s day-to-day lives. The proportion of Internet users over the age of 60 has climbed from 1.9% in 2010 to 11.3% in 2022, according to the 25th and 50th China Internet Development Statistics Reports published by the China Internet Network Information Center in 2010 and 2022, respectively. In the context of aging and the digital economy, how to improve the subjective well-being (SWB) of the elderly through the Internet has become an important topic.

Until now, SWB has different meanings in different fields. For instance, in the field of sociology, scholars have proposed “social well-being” and “psychological well-being,” which are expressions that focus more on satisfaction and identity in social activities (Winkelmann, 2008). Correspondingly, economists equate SWB with maximum utility based on the assumption of “rational people.” As Diener et al. (1999) highlighted that SWB was an individual’s emotional reflection, which essentially reflected the individual’s overall judgment of life satisfaction, physical satisfaction, and job satisfaction. In summary, SWB is a concept with multiple dimensions (Zhong et al., 2022) and it is worth paying more attention to SWB of the elderly from a multidimensional viewpoint.

Research on the Internet and elderly individuals’ SWB was first widely concerned in the field of psychology. Some scholars point out that using social media helps to build and strengthen people’s social connections and reduce their feelings of isolation and loneliness (Nam et al., 2019; Szabo et al., 2019; Lam et al., 2020; Meshi et al., 2020). The elderly with a higher frequency of social media use have better physical and mental health (Bianchi, 2021; Early and Hernandez, 2021; Nakagomi et al., 2022). In particular, the COVID-19 pandemic has reinforced the role of the Internet as the key determinant of reducing loneliness during the social-distancing orders (Hajek and König, 2021). Some studies, however, tell a different story. For example, Schemer et al. (2021) examined the average longitudinal impact of frequency of the Internet and social networking sites (SNS) on SWB of adolescents in Germany, and found that the frequency of the Internet in general and SNS in particular was not substantially related SWB. Sabatini and Sarracino (2017) verified the negative impact of the Internet on SWB from the life domain. They concluded that the use of the Internet reduced the frequency and quality of face-to-face communication between family members, leading to a deterioration in family relationships and a decrease in life satisfaction. In addition, problematic Internet use (e.g., addiction or compulsive Internet use) negatively affects SWB (Nie, 2001; Casale and Fioravanti, 2011; Brooks, 2015). Overall, there is no consensus on whether the Internet contributes to elderly individuals’ SWB.

Although the literature on the Internet and elderly individuals’ SWB serves as a good guide for this study, previous studies usually depict SWB using one-dimensional metrics, such as life satisfaction or loneliness (Li and Zhou, 2021; Lu and Kandilov, 2021; Tang et al., 2022). From a multidimensional perspective, Zhong et al. (2022) found that the Internet had a positive impact on residents’ job satisfaction, happiness, social relationships, and future confidence, but they focused on the adult group and did not explore the underlying mechanisms in depth. In particular, elderly individuals are away from work and are more concerned about their physical satisfaction and life satisfaction (Cotten et al., 2013; Zhang et al., 2021). However, as an important influencing factor of SWB of the elderly, existing studies still pay little attention to the relationship between the Internet and physical satisfaction of the elderly. To bridge the research gap, this study empirically examines the effects of the Internet on the physical satisfaction and life satisfaction among the elderly based on CGSS, as well as identifying the underlying mechanism on the basis of the relevant theories.

Compared with the existing literature, we contribute to literature in several important ways. First, this study contributes to the current research related on the factors influencing SWB. Since the introduction of the Easterlin paradox, how to increase SWB is a recent issue of academic interest, covering financial level, educational experience, social consciousness, etc. (Easterlin et al., 2010; Prati et al., 2016; Lu and Kandilov, 2021). This study defines elderly individuals’ SWB in terms of different dimensions and incorporates them into an analytical framework to describe SWB in greater depth. From this perspective, this study finds that Internet use has a positive effect on the physical satisfaction and life satisfaction of elderly individuals, which provides new empirical evidence that the Internet affects SWB of elderly individuals in developing countries.

Second, this study expands the micro evidence on how the Internet affects elderly individuals’ SWB in the Chinese setting. Although it has been shown that social activities are an important factor affecting life satisfaction (Burke et al., 2010; Oh et al., 2014; Castellacci and Tveito, 2018), as an important factor in non-western cultures, there is little research available on the impact of how the Internet affects kin ties and friend ties to enhance elderly individuals’ life satisfaction. The Internet can break down communication barriers in time and space and help elderly individuals build social connections and facilitate a higher quality of their face-to-face relationships, which strengthens family ties and expands their non-family ties (Yu et al., 2016). Meanwhile, the Internet provides an online social environment for the elderly (Sinclair and Grieve, 2017) and helps elderly individuals communicate with each other and strengthen their relationships with friends. In addition, spending time online can enrich elderly individuals’ leisure activities and satisfy their need for social contact with the outside world, contributing to their life satisfaction (Choi and Lehto, 2009; Zhou and Peng, 2018). Based on CGSS, this study not only confirms the positive effect of kins ties and friend ties on the increase of life satisfaction of the elderly, but also confirms the positive significance of social activities.

At the same time, considerable evidence shows that health insurance plays an important role in improving self-reported health status, reducing mortality risk (Braveman and Gruskin, 2003; Hadley and Waidmann, 2006; Cheng et al., 2015; Fan et al., 2020), reducing medical expenditure and financial burden (Batty et al., 2022), while it is still unknown whether the Internet can help to enhance health insurance and further improve their physical satisfaction. Compared with traditional communication channels, the Internet has advantages in information exchanging and sharing functions (Li and Zhou, 2021), which reduces information asymmetry between consumers and producers, and reduces online search costs for consumers (Brown and Goolsbee, 2002). By using big data to study elderly individuals’ behavior and tastes, the Internet may create insurance products that effectively cater to the needs of various consumers and help supply and demand match. Moreover, the Internet helps the elderly to access and learn more about social security projects, which improves their awareness of social security projects. Therefore, this study confirms that health insurance is an important mechanism by which the Internet affects elderly individuals’ physical satisfaction.

Finally, this study uses various robustness tests such as the instrumental variables method and different Internet identification to further ensure the reliability of our findings. Moreover, in a practical sense, exploring the effects of the Internet on their life and physical satisfaction does provide useful references to increase SWB of the elderly and facilitate their adaption to technological changes and integration into the digital era.

The rest of the study is organized as follows. Section Data source and variable measurements describes the data source and variable measurements. Section Empirical analytic strategies and estimation results presents the results of the empirical analyses. Section Conclusion and implications concludes the study with a brief discussion.

## Data source and variable measurements

### Sample

The dataset in this study is mainly from the CGSS, covering the years 2010, 2011, 2012, 2013, 2015, and 2017. CGSS is the earliest nationwide, comprehensive and continuous academic survey project in China, with a very wide range of districts. CGSS not only contains the variables related to Internet use we are concerned about, but also includes relevant information at individual and social levels.

Given the purpose of the research, we selected the elderly residents who are >60 years old and <90 years old as the subjective of our research and removed the invalid samples. After eliminating the questionnaires that contain numerous missing information, we obtain 16,000 valid samples. Moreover, to ensure the robustness of the conclusions, this study also applies the CHARLS data released by Peking University from 2011 to 2018, with a sample size of 19,040.

### Measures

#### Dependent variables

This study focuses on the over 60s. In general, the two aspects that the elderly are most concerned about are the degree of satisfaction in physical health and life. Based on this, this study comprehensively develops SWB of the elderly from two aspects: physical satisfaction and life satisfaction.

Among them, respondents were asked to answer, “In general, do you think your life is happy?” and choose a value between 1 and 5 ranging from low life satisfaction to high life satisfaction, where 5 means “my life is very happy,” 4 means “my life is relatively happy,” 3 means “my life is generally happy,” 2 means “my life is not so happy,” and 1 means “my life is totally not happy at all,” respectively. Similarly, the respondents’ physical satisfaction is evaluated by asking them, “How do you think your current physical health is?” and choosing a value between 1 and 5 ranging from low physical satisfaction to high physical satisfaction, where 5 means “my current physical condition is very healthy,” 4 means “my current physical condition is relatively healthy,” 3 means “my current physical condition is generally healthy,” 2 means “my current physical condition is not so healthy,” and 1 means “my current physical condition is not healthy at all,” respectively.

#### Key independent variables

The main explanatory variable in this study is Internet use. In the survey questionnaire, respondents were asked about Internet use. The question we used was “In the past year, how often do you use the Internet?.” The answers ranged from 1 to 5 representing frequencies of Internet use from low to high, where 1 means “never use,” 2 means “barely use,” 3 means “sometimes use,” 4 means “often use,” and 5 means “very often use.”

#### Other control variables

First, personal control variables include work, income, gender, age, ethnicity, political status, marital status, and residence if one lives in rural area then the value is 1, and a set of province and time fixed effects. These basic individual characteristic variables shape a person’s cognitive abilities, personality, values, and attitudes, and they should partially explain the variation in SWB. Second, control variables that reflect the family situation of elderly individuals are considered. They include the number of sons and daughters in the family, and family wealth (whether the household owns a house). Third, the social level control variables include the degree of social trust (a scale variable from low to high social trusting valuing 1 to 5), and the degree of social fairness (a scale variable from low to high social fairness valuing 1 to 5). The specific variable descriptions are shown in Table 1.

**Table 1:** Variable description and descriptive statistics.

Variable name	Variable meaning	Obs	Mean	SD	Min	Max
Life satisfaction	Elderly individuals’ life satisfaction	16,000	3.88	0.85	1	5
Physical satisfaction	Elderly individuals’ physical satisfaction	16,000	3.09	1.10	1	5
Internet	Degree of internet use	16,000	1.36	1.05	1	5
Work	Working status	16,000	0.297	0.46	0	1
Income	Logarithmic personal annual income	16,000	7.498	3.72	0	9.81
Residence	Urban residents = 0, rural residents = 1	16,000	0.48	0.50	0	1
Ethnicity	Minority = 0, Han = 1	16,000	0.93	0.25	0	1
Political status	Others =0, communist = 1	16,000	0.29	0.45	0	1
Marital status	Unmarried = 0, Married = 1	16,000	0.71	0.45	0	1
Age	Age in the survey year	16,000	69.78	6.78	60	90
Gender	Female = 0 male = 1	16,000	0.51	0.50	0	1
Sons	Number of sons in the family	16,000	1.50	1.06	0	8
Daughters	Number of daughters in the family	16,000	1.30	1.16	0	7
Family wealth	If own, value is 1; if not, value is 0	16,000	0.93	0.25	0	1
Social trust	Low social trust:1, high social trust:5	16,000	3.30	1.03	1	5
Social fairness	Low social fainess:1, high social fairnesst:5	16,000	3.62	0.97	1	5

## Empirical analytic strategies and estimation results

### Empirical model

Considering the data characteristics of the explained variables, we referred to Zhou et al. (2022) and ran Ordered Probit model to analyze and estimate the effect of the Internet on SWB of the elderly. The model settings are as follows:


(1) $$SWB_{ijt}=f\;\left(\mathrm{Interne}t_{ijt},X_{ijt}\right)+\varepsilon_{ijt}\text{.}$$

where SWB represents the physical satisfaction and life satisfaction of the elderly *i* who living in province *j* in year *t*, considering a value between 1 and 5 measuring levels. Internet use is the core explanatory variable, representing the degree of Internet use of the elderly *i*, ranging from 1 to 5. X is the vector of control variables including individual-level, household-level, social-level control variables above, and fixed effects of province and time. ε represents a random error term.

The method of step-test regression coefficient is commonly used to test the mediation effect. Therefore, we adopt the step-test regression coefficient method to examine how the Internet affects physical satisfaction and life satisfaction. Mediation represents health insurance and social networks. The specific model is constructed as follows:


(2) $$\mathrm{Mediatio}n_{ijt}=\alpha_{0}+\alpha_{1}\mathrm{Interne}t_{ijt}+\alpha_{n}X_{n}+\delta_{j}+\gamma_{t}+\varepsilon_{ijt}$$

(3) $$\begin{array}{c} SWB_{ijt}=\beta_{0}+\beta_{1}\mathrm{Interne}t_{ijt}+\beta_{2}\mathrm{Mediatio}n_{ijt}+\\ \beta_{n}X_{n}+\delta_{j}+\gamma_{t}+\varepsilon_{ijt} \end{array}$$

### Benchmark regression results

In this section, we ran the Ordered Probit method to test the relationship between the Internet and SWB of the elderly by gradually adding control variables. The results are shown in Table 2. The results in columns 1 and 4 do not include other control variables, and then in columns 2 and 5 the control variables and fixed effects are included. Given the connection between life satisfaction and physical satisfaction (Kesavayuth et al., 2022), we added each of them as control variables to the regression equation. The results indicate that after controlling the characteristics of elderly individuals and fixed effects, the impact of the Internet on the life satisfaction and physical satisfaction of elderly individuals are significantly positive. It has initially demonstrated the point in the relevant literature that the Internet affects SWB of the elderly. On the one hand, the Internet can enhance the efficiency of elderly individuals in receiving and feeding back information (Purcell et al., 2013), which expands the scope of social communication and promotes the diversification of communication patterns and social interactions. On the other hand, elderly individuals can benefit from the diagnosis, treatment, and monitoring of diseases through telemedicine (Doica et al., 2021), which guarantees physical satisfaction. At the same time, to eliminate the worry of the problem of model misdesign, we ran the OLS model to test the effect of the Internet on the life satisfaction and physical satisfaction of elderly individuals again (columns 3 and 6 of Table 2). The results show that at the 1% level of significance, an increase of 1 unit in Internet use improves life satisfaction by 0.0186 points on a 5-point scale, and physical satisfaction by 0.0559 point. These results further highlight the objective fact that the Internet increases the life satisfaction and physical satisfaction of elderly individuals. Overall, although previous studies on the effect of the Internet on SWB have produced inconclusive, we provide evidence that Internet use has a positive effect on SWB of elderly individuals in China.

**Table 2:** Benchmark regression results.

Variables	Ordered probit		OLS	Ordered probit		OLS
	(1)	(2)	(3)	(4)	(5)	(6)
	Life satisfaction			Physical satisfaction		
Internet	0.0488^***^ (6.3849)	0.0232^**^ (2.5101)	0.0186^***^ (3.1801)	0.0990^***^ (14.0814)	0.0561^***^ (6.8631)	0.0559^***^ (7.1133)
Physical satisfaction		0.2312^***^ (24.9149)	0.1602^***^ (25.3601)			
Life satisfaction					0.2982^***^ (25.6443)	0.2837^***^ (26.6947)
Work		−0.0958^***^ (−4.0762)	−0.0625^***^ (−3.7947)		0.1977^***^ (8.8771)	0.1895^***^ (8.8839)
Income		0.0131^***^ (4.6367)	0.0094^***^ (4.7567)		0.0154^***^ (5.6048)	0.0149^***^ (5.6151)
Residence		0.1628^***^ (6.5574)	0.1120^***^ (6.5723)		0.1715^***^ (7.2623)	0.1658^***^ (7.2893)
Ethnicity		−0.0957^**^ (−2.2795)	−0.0644^**^ (−2.2890)		−0.1114^***^ (−2.7118)	−0.1014^***^ (−2.5772)
Political status		0.1188^***^ (4.9531)	0.0791^***^ (5.2399)		0.0210 (0.9261)	0.0211 (0.9702)
Marital status		0.2162^***^ (10.0203)	0.1519^***^ (10.1875)		0.0667^***^ (3.2951)	0.0635^***^ (3.2553)
Age		0.0056^***^ (3.4712)	0.0030^***^ (2.7064)		−0.0108^***^ (−7.1369)	−0.0100^***^ (−6.8825)
Gender		0.1277^***^ (6.7335)	0.0801^***^ (6.2354)		0.2004^***^ (11.2153)	0.1935^***^ (11.2495)
Sons		0.0503^***^ (5.0025)	0.0346^***^ (4.9731)		−0.0258^***^ (−2.7633)	−0.0257^***^ (−2.8592)
Daughters		0.0534^***^ (6.0822)	0.0358^***^ (5.8711)		−0.0285^***^ (−3.5056)	−0.0272^***^ (−3.4727)
Family wealth		0.0649^*^ (1.7536)	0.0520^**^ (2.0179)		0.0582^*^ (1.7736)	0.0532^*^ (1.6651)
Social trust		0.3448^***^ (32.4967)	0.2371^***^ (32.6876)		−0.0057 (−0.6063)	−0.0042 (−0.4662)
Social fairness		0.1142^***^ (10.8219)	0.0740^***^ (10.0572)		0.0212^**^ (2.2031)	0.0212^**^ (2.3141)
Province FE	NO	YES	YES	NO	YES	YES
Year FE	NO	YES	YES	NO	YES	YES
Observations	16,000	16,000	16,000	16,000	16,000	16,000
R^2^			0.1983			0.1448
Pseudo R^2^	0.0011	0.0966		0.0033	0.0538	

*** Mean significant at 1% level.

** Mean significant at 5% level.

* Mean significant at 10% level.

*t*-Values in bracket is clustered.

In terms of control variables, personal characteristics and family factors also influence the physical satisfaction and life satisfaction of elder individuals. Income, residence, ethnicity, political status, gender, marital status, and family wealth all produce the same effect on SWB. Although work squeezes the leisure time of the elderly and brings a negative impact on the life satisfaction, the medical checkups provided by the workplace bring health protection to the elderly. In terms of age, elderly individuals have more leisure time to dominate their lives, which is an important way to improve life satisfaction (Zhou and Peng, 2018). At the same time, as age increases, elderly individuals have more diseases, leading to a negative effect of age on physical satisfaction. As for family factors, in traditional Chinese families, elderly individuals take on the responsibility of raising young children. On the one hand, young children bring joy to the lives of elderly individuals. On the other hand, elderly individuals need to spend more time and energy taking care of young children, which adds to the physical burden. Aspects of social factors, social trust and social fairness both exert a positive impact on life satisfaction and physical satisfaction.

**Figure 1:**
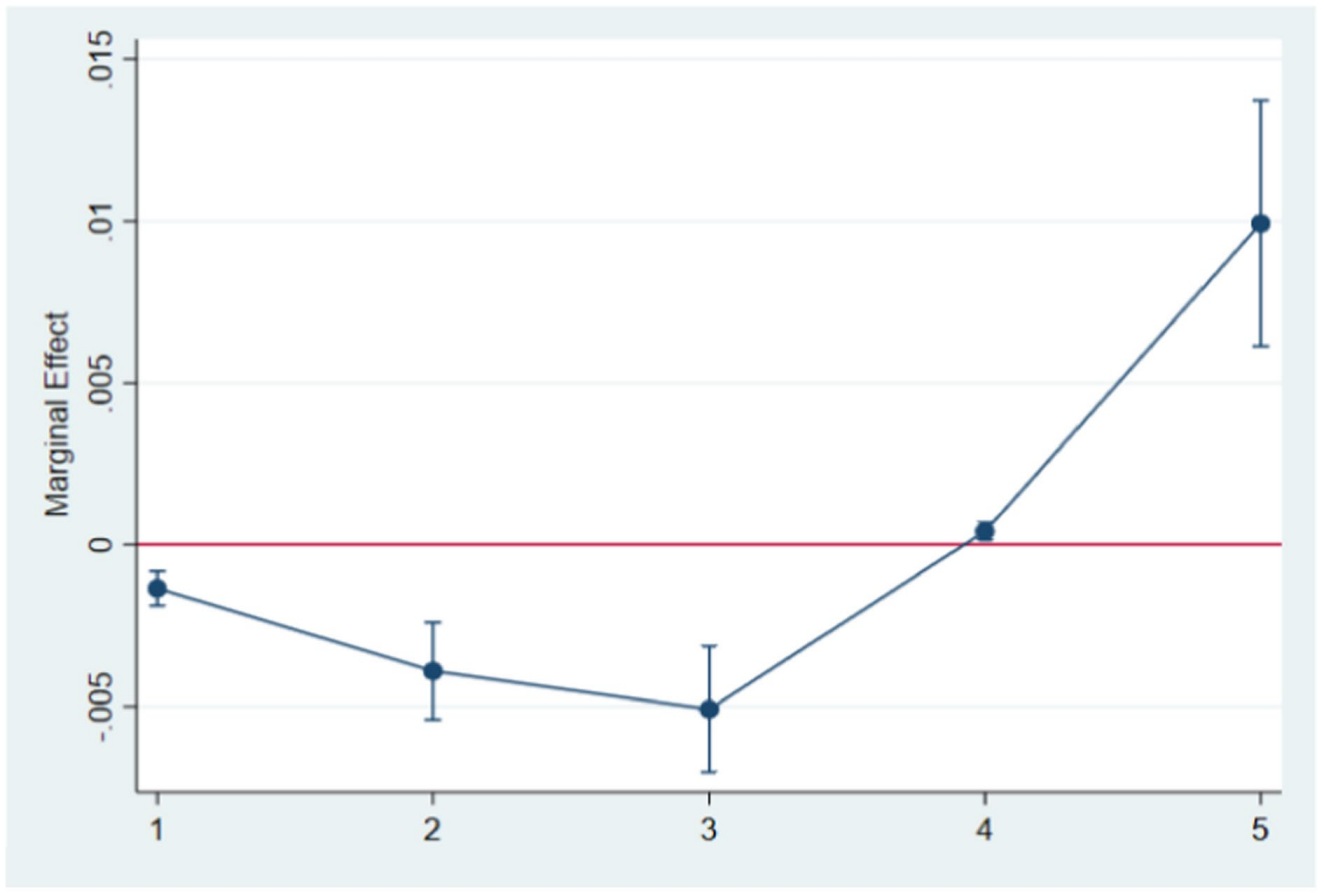
The effect of Internet use on life satisfaction.

**Figure 2:**
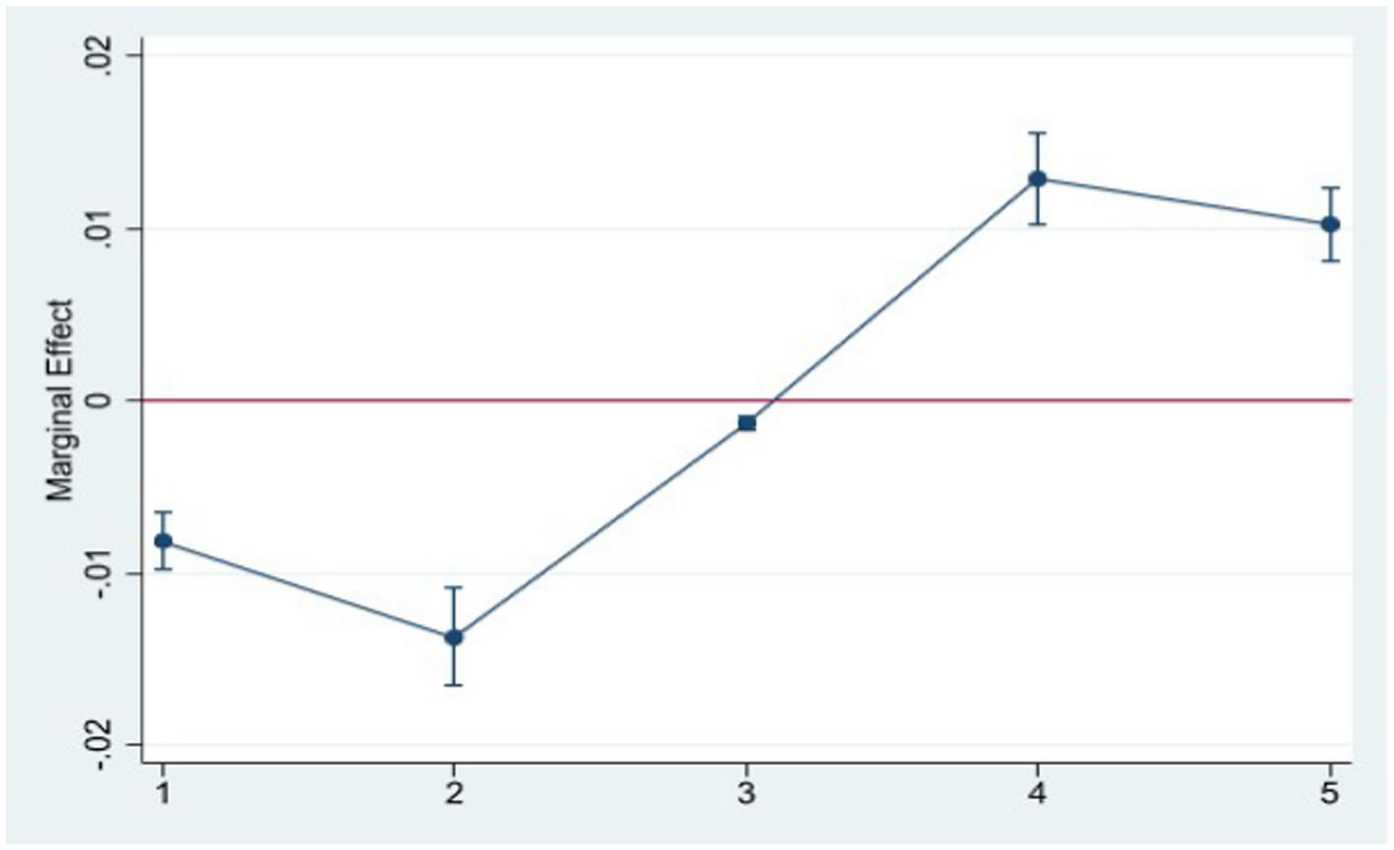
The effect of Internet use on physical satisfaction.

Moreover, to make the article more concise, we presented the marginal effects of Internet use on various life and physical satisfaction scores (1–5) in the form of graphs. As shown in Figures 1, 2, Internet use can effectively reduce the probability of the elderly feeling unsatisfaction in life and physical and increase the probability of feeling satisfied.

### Robustness check

To further ensure the robustness of the benchmark results, we used different Internet use identification and database, instrumental variable methods, and various interference factors to deal with potential problems.

### Different identification and database

First, replace the explanatory variable of Internet use. We selected the questions “Is the Internet your main source of information in the past year?,” and “In the past year, how often you spent your free time online?” as the substitution variables for the degree of Internet use. Columns (1) to (4) of Table 3 show the results after changing the explanatory variables, and the results show that the use of the Internet by elderly individuals still contributes to health satisfaction and life satisfaction, which is consistent with benchmark regression.

**Table 3:** Robustness test.

Variables	Changing the explanatory variables				Changing database	
	(1)	(2)	(3)	(4)	(5)	(6)
	Life satisfaction	Physical satisfaction	Life satisfaction	Physical satisfaction	Life satisfaction	Physical satisfaction
Internet (0–1)	0.0880^***^ (3.1540)	0.2192^***^ (8.5181)				
Internet (1–5)			0.0145^*^ (1.7636)	0.0309^***^ (3.1346)		
Internet					0.2958^***^ (5.8511)	0.5861^***^ (7.2631)
Controls	YES	YES	YES	YES	YES	YES
Province FE	YES	YES	YES	YES	YES	YES
Year FE	YES	YES	YES	YES	YES	YES
Observations	16,000	16,000	16,000	16,000	19,040	19,040
Pseudo R^2^	0.0771	0.0543	0.0965	0.0535	0.0142	0.0245

*** Mean significant at 1% level.

** Mean significant at 5% level.

* Mean significant at 10% level.

*t*-Values in bracket is clustered. All regression controls for all variates as regression of Table 2. The table below demonstrates the same.

Second, replace the database. We used the Charls database released by Peking University for the fourth period from 2011 to 2018 to retest. According to the questions in the questionnaire design, we choose “Generally speaking, are you satisfied with your life?,” and “Generally speaking, are you satisfied with your health?,” which score from low to high on a scale of 1–5. Columns (5) to (6) of Table 3 show that the impacts of Internet use on SWB of elderly individuals are still significantly positive.

### Instrumental variable methods

Endogeneity may cause deviations and affect the estimation results, such as potential unobserved factors, reverse causality problems, etc. To deal with endogenous issues, we then resorted to the instrumental variable approach. Based on the existing literature, we took the historical telephone and post office penetration data of the city in 1984 as the instrumental variables of individual Internet use. The specific reason for this is that, on the one hand, areas with high historical telephone and post office penetration rates are very likely to have a high level of information exchange, which is highly related to Internet access. On the other hand, historical data has little effect on the current elderly individuals’ SWB, which is in line with the exogenous principle. It should be noted that since historical data is a time-invariant variable, which may be absorbed by the fixed effect. Therefore, referring to Nunn and Qian (2014), we multiplied the number of post offices and the number of telephones in the number of Internet users nationwide in the previous year as an instrumental variable for the use of the Internet by the elderly.

Based on this, we further used the IV-oprobit model to test the relationship between Internet use and SWB of the elderly again. Table 4 reports the results. The results show that there is an obvious positive correlation between the instrumental variables and Internet use, which means that they meet the correlation hypothesis. The coefficient of Internet use is still significantly positive. Moreover, the model passes the atanhrho_12 test, indicating that the instrumental variables constructed in this study are effective. This result is consistent with the result of benchmark testing.

**Table 4:** Endogenous processing: IV-oprobit.

Variables	(1)	(2)	(3)	(4)
	Internet	Life satisfaction	Internet	Physical satisfaction
Post office penetration	0.4041^***^ (14.4312)		0.4010^***^ (14.2521)	
Historical phone	1.1451^***^ (18.8325)		1.1497^***^ (18.9231)	
Internet		0.0466^***^ (4.5314)		0.0276^**^ (1.9651)
Controls	YES	YES	YES	YES
Province FE	YES	YES	YES	YES
Year FE	YES	YES	YES	YES
Observations	16,000	16,000	16,000	16,000
atanhrho_12		0.02627^**^		0 0.06241^**^

*** Mean significant at 1% level.

** Mean significant at 5% level.

* Mean significant at 10% level.

*t*-Values in bracket is clustered.

### Other robustness tests

In this section, several other processing methods are carried out to eliminate as far as possible factors that affect the reliability of the results. The first is to eliminate policy interference. To alleviate Internet application problems such as slow network speed and low coverage, China implemented the “Broadband China” strategy in August 2013, which specifies that China will take a regional pilot approach to build and improve broadband infrastructure. The first batch of pilot cities appeared in 2014, and a total of 39 cities (city clusters), including Beijing, Shanghai, and Guangzhou. The second and third batches appeared in 2015 and 2016, respectively. “Broadband China” strategy is of great significance to the popularization of the Internet (Wan et al., 2021). Therefore, we excluded research samples after 2014. The regression results indicate that after excluding the post-2014 sample, the positive effect of the Internet on elderly individuals’ SWB remains.

The second is to eliminate the interference of municipalities directly under the central government. In fact, municipalities directly under the central government have great economic and political particularity. The fiber optic network, Internet penetration rate, and actual user experience of Internet access in the municipalities are significantly higher than those in other provinces. In this regard, we excluded the sample of municipalities directly under the central government. Combined with the results in columns 3 and 4 of Table 5, after excluding the sample of municipalities directly under the central government, Internet use still positively affects life satisfaction and physical satisfaction of elderly individuals.

**Table 5:** Other robustness tests.

Variables	(1)	(2)	(3)	(4)	(5)	(6)
	Eliminate policy interference		Eliminate the interference of municipalities		Eliminate sampling error	
	Life satisfaction	Physical satisfaction	Life satisfaction	Physical satisfaction	Life satisfaction	Physical satisfaction
Internet	0.0294^**^ (1.9627)	0.0467^***^ (3.7084)	0.0440^***^ (3.4894)	0.0665^***^ (5.8260)	0.0305^**^ (2.4151)	0.0698^***^ (6.1829)
Controls	YES	YES	YES	YES	YES	YES
Province FE	YES	YES	YES	YES	YES	YES
Year FE	YES	YES	YES	YES	YES	YES
Observations	9,224	9,224	12,928	12,928	10,242	10,242
Pseudo R^2^	0.0804	0.0539	0.0968	0.0528	0.0940	0.0557

*** Mean significant at 1% level.

** Mean significant at 5% level.

* Mean significant at 10% level.

*t*-Values in bracket is clustered.

The third is to eliminate the interference of sampling error of related samples. Statistical results may bias estimates when sampling is uneven across regions (Alfaro et al., 2016). According to the distribution of CGSS sampling areas, we deleted six regions with a sampling proportion of less than 1%, including Hainan Province, Qinghai Province, etc. At the same time, we also deleted six regions with a sampling proportion higher than 5%, including Henan Province, Hubei Province, Sichuan Province, etc. The results in columns 5 and 6 in Table 5 show that the coefficients of Internet use are consistent and significantly positive.

### Mechanism analysis

#### Physical satisfaction mechanism analysis

Studies focusing on elderly individuals state that the increase of health insurance coverage for the elderly by medical care has improved the ability of individuals to resist the impact of diseases and the health level of individuals (Card et al., 2008; Finkelstein and Mcknight, 2008). To gain more insight into the relationship between Internet use and physical satisfaction, we utilized variables available in health insurance in our dataset. The intermediate variables are constructed from a set of three questions that evaluate elderly individuals’ participation in health insurance. The questions comprised: “Do you participate in the health insurance project?,” “Do you currently participate in commercial health insurance?” and “Do you currently participate in urban basic health insurance/new rural cooperative health insurance/public health?.” We coded the answers given by respondents for questions as 0 (no participate) or 1 (participate). The regression results are shown in Table 6.

**Table 6:** Physical satisfaction mechanism analysis.

Variables	(1)	(2)	(3)	(4)	(5)	(6)	(7)
	Physical satisfaction	Health insurance	Physical satisfaction	Commercial insurance	Physical satisfaction	Basic insurance	Physical satisfaction
Internet	0.0647^***^ (7.7412)	0.0330^**^ (2.3924)	0.0645^***^ (7.7297)	0.0500^***^ (2.7629)	0.0633^***^ (7.8056)	0.0241^*^ (1.6994)	0.0647^***^ (7.7459)
Health insurance			0.0297^*^ (1.8038)				
Commercial insurance					−0.0320 (−1.0111)		
Basic insurance							0.0598^***^ (2.7880)
Controls	YES	YES	YES	YES	YES	YES	YES
Province FE	YES	YES	YES	YES	YES	YES	YES
Year FE	YES	YES	YES	YES	YES	YES	YES
Observations	14,746	14,746	14,746	14,746	14,746	14,746	14,746
Pseudo R^2^	0.0334	0.0851	0.0301	0.0504	0.0334	0.1197	0.0335

*** Mean significant at 1% level.

** Mean significant at 5% level.

* Mean significant at 10% level.

*t*-Values in bracket is clustered.

According to the CGSS questionnaire results, some elderly individuals did not answer whether they participated in health insurance, which led to sampling bias in the mechanism analysis and overall regression. To ensure the reliability of the mechanism channel, we first examined the overall effect between Internet use and physical satisfaction of elderly individuals again. Column 1 in Table 6 presents that after processing the sample data size, Internet use remains positively significant on physical satisfaction. We further explored whether Internet use contributes to increasing the participation of elderly individuals in health insurance. The results reveal that conditional on other covariates, Internet use exerts a significant positive impact on health insurance, commercial health insurance, and basic health insurance (columns 2, 4, and 6). Then, we put the mediating variables in the regression to test the effect on physical satisfaction. Columns 3, 5, and 7 of Table 6 show that health insurance and basic health insurance exert a significant impact on physical satisfaction, while commercial health insurance does not.

#### Life satisfaction mechanism analysis

As documented in the literature, social networks play a vital role in an elderly individuals’ life satisfaction. Specifically, we have data on social activities, kin ties and friend ties. Social activities are measured on a 5-point scale based on the response to the question “How often do you socialize in your spare time?,” with 1 indicating “never” and 5 indicating “frequently.” Similarly, the measure of kin ties stems from a question that “How often do you gather with relatives who do not live together?,” with 1 indicating “never” and 5 indicating “frequently.” As for friend ties, respondents were asked to rate how often you have social entertainment with other friends on a 5-point scale from “never” 1 to “frequently” 5. The results are shown in Table 7.

**Table 7:** Life satisfaction mechanism analysis.

Variables	(1)	(2)	(3)	(4)	(5)	(6)	(7)
	Life satisfaction	Social activities	Life satisfaction	Friend ties	Life satisfaction	Kin ties	Life satisfaction
Internet	0.0232^**^ (2.5101)	0.0359^***^ (4.0625)	0.0206^**^ (2.2194)	0.0878^***^ (9.8065)	0.0206^**^ (2.2205)	0.0491^***^ (5.0432)	0.0260^***^ (2.5819)
Social activities			0.0813^***^ (9.3725)				
Friend ties					0.0312^*^ (1.9547)		
Kin ties							0.0150^**^ (2.5374)
Control	YES	YES	YES	YES	YES	YES	YES
Province FE	YES	YES	YES	YES	YES	YES	YES
Year FE	YES	YES	YES	YES	YES	YES	YES
Observations	16,000	16,000	16,000	16,000	16,000	13,252	13,252
Pseudo R^2^	0.0966	0.0223	0.0992	0.0336	0.0972	0.0114	0.0972

*** Mean significant at 1% level.

** Mean significant at 5% level.

* Mean significant at 10% level.

*t*-Values in bracket is clustered.

Column 1 in Table 7 presents the regression results from column 2 of Table 2, repeated here for convenience. The results reveal that Internet use exerts a significant positive impact on social activity, kin ties and friend ties (see columns 2, 4, and 6). Columns 3, 5, and 7 in Table 7 present estimates of the impact of Internet use on life satisfaction, conditional on social activities, friend ties and kin ties, respectively. The results show that all intermediate variables play a vital role in life satisfaction of elderly individuals. In summary, it is reasonable to conclude that the changes in social activities, friend ties and kin ties serve as important mechanisms through which the Internet positively affects life satisfaction among elderly individuals in China.

### Heterogeneity analysis

To examine the heterogeneous impact of the Internet on elderly individuals, we analyzed the impact of the Internet from three aspects: urban and rural areas, education level, and gender.

#### Urban–rural heterogeneity

Considering the inherent dual structure of urban and rural areas in China, Internet use may have different effects on SWB of elderly individuals in different regions. According to the household registration of elderly individuals, we divided elderly individuals into urban groups and rural groups (Table 8). The results show that Internet use has an important impact on life satisfaction and physical satisfaction among elderly individuals in different regions, and the subjective welfare effect of Internet use has a relatively larger effect on the urban elderly. This is partly due to the urban elderly individuals having an advantageous position in economic fields such as income, and being more likely to access and use the Internet (Pandey et al., 2003).

**Table 8:** Urban–rural heterogeneity.

	(1) Urban	(2) Rural	(3) Urban	(4) Rural
Variables	Life satisfaction		Physical satisfaction	
Internet	0.0593^**^ (1.9635)	0.0243^**^ (2.4018)	0.0855^***^ (3.4194)	0.0508^***^ (5.4863)
Controls	YES	YES	YES	YES
Province FE	YES	YES	YES	YES
Year FE	YES	YES	YES	YES
Observations	8,349	7,651	8,349	7,651
Pseudo R^2^	0.0959	0.0987	0.0526	0.0496

*** Mean significant at 1% level.

** Mean significant at 5% level.

* Mean significant at 10% level.

*t*-Values in bracket is clustered.

#### Individual heterogeneity

We divided elderly individuals into high human capital and low human capital groups according to whether elderly individuals have a high school degree and divided into female elderly group and male elderly group according to gender (Table 9). From the perspective of education structure, it is in line with the reality that Internet use has a greater impact on the life satisfaction and physical satisfaction of elderly individuals with higher education levels. The Internet is a skill-biased technology, and high educated individuals have stronger learning abilities and can better absorb the digital dividends brought by the use of the Internet.

**Table 9:** Individual heterogeneity.

Variables	Different education structure group			
	(1) Low capital	(2) High capital	(3) Low capital	(4) High capital
	Life satisfaction		Physical satisfaction	
Panel A				
Internet	0.0315^**^ (2.4064)	0.0487^***^ (3.2382)	0.0798^***^ (6.8834)	0.1351^***^ (2.6066)
Controls	YES	YES	YES	YES
Province FE	YES	YES	YES	YES
Year FE	YES	YES	YES	YES
Observations	13,373	2,627	13,373	2,627
Pseudo R^2^	0.0780	0.0803	0.0352	0.0415
**Variables**	**Different gender groups**			
	**(1) Female**	**(2) Male**	**(3) Female**	**(4) Male**
	**Life satisfaction**		**Physical satisfaction**	
Panel B				
Internet	0.0025 (0.1840)	0.0472^***^ (3.7328)	0.0425^***^ (3.4621)	0.0651^***^ (5.8377)
Controls	YES	YES	YES	YES
Province FE	YES	YES	YES	YES
Year FE	YES	YES	YES	YES
Observations	7,893	8,107	7,893	8,107
Pseudo R^2^	0.0942	0.1019	0.0472	0.0574

*** Mean significant at 1% level.

** Mean significant at 5% level.

* Mean significant at 10% level.

*t*-Values in bracket is clustered.

From the perspective of gender, results show that Internet use has greater effect on the improvement of males’ life satisfaction and physical satisfaction, which is consistent with Lu and Kandilov (2021). Compared to males, females perform more household services, including intergenerational support, and caring for relatives. Females, meanwhile, are more sensitive, vulnerable, and less able to withstand it than men.

## Conclusion and implications

In the dual context of a deepening aging population and a brand new change in the digital age, the impact of the Internet on elderly individuals has become a hot topic of interest. To our knowledge, most of the existing literature focuses on the overall effect of both (Lu and Kandilov, 2021; Tang et al., 2022), lacking comprehensive consideration of SWB of the elderly. In this study, we explored the different impacts of Internet use on elderly individuals’ SWB and analyzed the potential mechanism from the perspective of physical and life satisfaction by using nationally representative data from China (CGSS). This study is a useful addition to existing research on the factors and potential mechanisms influencing SWB in elderly individuals and provides a reliable basis for the government to develop precise Internet policies for elderly individuals.

We first explored the role played by the Internet on life satisfaction and physical satisfaction of elderly individuals in China empirically. Meanwhile, we addressed the issue of endogeneity by running a two-stage least square estimation with historical data as instrumental variables, and used a series of robustness tests to ensure valid results. Estimates consistently suggest that there exist significant positive effects of the Internet on life satisfaction and physical satisfaction of elderly individuals. These results highlight the important micro effects of the Internet and underscore the critical role the Internet plays in promoting SWB of elderly individuals in China.

We further considered potential mechanisms for how the Internet may affect life satisfaction and physical satisfaction of elderly individuals. On the one hand, the Internet helps elderly individuals learn more about the Medicare program and increases health insurance participation rates of the Medicare program, which is critical to their physical satisfaction. On the other hand, we found that the Internet can help to promote social activities in spare time and enhance relationships between elderly individuals and others, which we interpret as potential mechanisms behind the positive nexus between the Internet and life satisfaction among elderly individuals in China. Moreover, in terms of heterogeneity analysis, the beneficial impacts of the Internet on life satisfaction and physical satisfaction are larger for elderly individuals in urban areas, high human capital, and older male individuals. These results reflect the continued existence of urban–rural, gender, and education digital divide in China.

Finally, several shortcomings of this research warrant discussion. First, although the robustness check shows the validity of the effect, we cannot rule out the possibility of all potential endogenous concerns, which may lead to biased results. Future research should be conducted by finding appropriate external shocks whenever possible. Second, limited by the availability of questionnaire data, we only verify the underlying mechanism of the Internet affecting physical satisfaction and life satisfaction, while we do not further discuss the internal mechanism of individual heterogeneity. In this regard, future research should show the heterogeneous influence of individuals and comprehensively capture the internal channels through which the Internet affects SWB of elderly individuals at different levels, such as the structure of education and the regional structure.

## Data availability statement

The original contributions presented in the study are included in the article/supplementary material, further inquiries can be directed to the corresponding author.

## Ethics statement

Ethical review and approval was not required for the study on human participants in accordance with the local legislation and institutional requirements. The patients/participants provided their written informed consent to participate in this study.

## Author contributions

SM contributed to the writing—original draft, methodology, visualization, and resources. DC contributed to the writing review and editing, methodology, and formal analysis. All authors contributed to the article and approved the submitted version.

## Conflict of interest

The authors declare that the research was conducted in the absence of any commercial or financial relationships that could be construed as a potential conflict of interest.

## Publisher’s note

All claims expressed in this article are solely those of the authors and do not necessarily represent those of their affiliated organizations, or those of the publisher, the editors and the reviewers. Any product that may be evaluated in this article, or claim that may be made by its manufacturer, is not guaranteed or endorsed by the publisher.
